# 多发性原发性肺癌的治疗进展

**DOI:** 10.3779/j.issn.1009-3419.2025.102.17

**Published:** 2025-06-30

**Authors:** Ying XIAO, Xiaobo CHEN, Xinghe TONG, Xudong YANG, Yanlong YANG, Yunping ZHAO

**Affiliations:** 650100 昆明，昆明医科大学第一附属医院胸外科; Department of Thoracic Surgery, The First Affiliated Hospital of Kunming Medical University, Kunming 650100, China

**Keywords:** 肺肿瘤, 多模式治疗, 手术, 消融, 放疗, 药物治疗, Lung neoplasms, Multimodal therapy, Surgery, Ablation, Radiation therapy, Drug therapy

## Abstract

近年来，多发性原发性肺癌（multiple primary lung cancer, MPLC）不断增加，在临床工作中不容忽视。MPLC的治疗方式目前仍有争议，但手术被公认为最主要的治疗方式，目前研究表明，MPLC的治疗需根据不同的患者制定多模式治疗。本文综述了MPLC的多种治疗方法，包括手术、消融、化疗、放疗、靶向治疗和免疫治疗，旨在促进对MPLC治疗的认识。

## Abstract (en)

In recent years, the incidence of multiple primary lung cancer (MPLC) has been increasing, and it cannot be ignored in clinical practice. The treatment of MPLC is still controversial, but surgical treatment is recognized as the most important treatment. However, current studies have shown that the treatment of MPLC needs to develop multimodal treatment according to different patients. This review summarizes multiple treatment method for MPLC, including surgery, ablation, chemotherapy, radiotherapy, targeted therapy, and immunotherapy in order to enhance understanding of MPLC treatment.

多发性原发性肺癌（multiple primary lung cancer, MPLC）指同一患者在肺的一个或不同部位，同时或先后发生两个或两个以上的原发性肺癌病灶。MPLC的发病率正逐年攀升，2000年前为0.2%-3.4%，2000年后升至0.3%-8.0%。值得注意的是，国内外MPLC发病率存在差异，国外发病率为0.20%-8.00%，国内为0.3%-3.44%，略低于国际水平^[1]^。

适宜手术的I-III期非小细胞肺癌（non-small cell lung cancer, NSCLC）群体中，约有16%的患者术前影像学检查被检出存在多发结节病灶，有1%-8%被诊断为MPLC^[1]^。针对MPLC的治疗方案，目前医学界尚未达成普遍共识，但国内外相关文献在MPLC的治疗原则上持相同见解，即主张以外科手术为主导，辅以消融技术、放化疗等多学科协同的治疗路径。

## 1 手术治疗

当前针对MPLC的首选治疗方式为手术切除，其核心理念为在尽可能维护正常肺组织的同时，彻底切除肿瘤组织。根据结节的位置和数量，可以选择肺叶切除、肺段切除或楔形切除等手术方式^[2]^。

MPLC患者接受亚肺叶切除术生存质量明显优于肺叶切除术，手术方式更推荐亚肺叶切除术。一项针对全肺切除术与亚肺叶切除术效果的研究^[3]^共纳入26例同时性MPLC（synchronous MPLC, sMPLC）手术患者，结果显示，接受全肺切除术的患者生存质量欠佳，而接受亚肺叶切除术的患者表现出更佳的生存质量。在另一项对97例sMPLC患者进行生存分析的回顾性研究中^[4]^，无论选择亚肺叶切除术还是肺叶切除术，两组在5年总生存（overall survival, OS）率及5年无进展生存（progression-free survival, PFS）率方面没有明显差异。此外，JCOG0802试验^[5]^揭示了对于直径<2 cm的N0期MPLC患者，肺段切除术在5年OS率方面优于肺叶切除术（94.3% vs 91.1%, *P*<0.001）。该试验表明，对于周围型肺癌、实性成分占比（consolidation tumor ratio, CTR）>0.5、最大直径≤2 cm的患者，肺段切除术是首选手术方式。

在选择MPLC的手术方式时，应考虑不同病灶的具体情况，手术时应先处理主病灶，较大结节行肺叶切除，小病灶行亚肺叶切除，若多发结节位于不同肺叶且直径<2 cm，可优先选择在不同肺叶中行肺段切除^[6]^。对于同一肺叶的MPLC，通常选择肺叶切除；而对于同侧肺不同肺叶的情况，较大的病变会选择肺叶切除，小病灶进行亚肺叶切除^[7]^。对于双侧肺的MPLC，多采用分期手术，先切除对预后影响较大、分期较晚的病变，患者康复后切除另一侧的病变，分期手术之间的时间间隔通常约为1个月^[8]^。如果病灶位于肺叶的边缘，楔形切除术可以有效地移除肿瘤，同时保留尽可能多的健康肺组织^[9]^。如果肿瘤较大或位于肺叶的中心，可能需要进行肺叶切除术，可以移除整个受影响的肺叶以及相关的淋巴结。

除目前常规单侧胸腔镜术式外，对于患有双肺多发结节的患者，进行同期双侧单孔胸腔镜手术是一种安全可行的治疗方式^[10,11]^。在一项进行同期双侧胸腔镜手术的研究^[10]^中，40例患者共计切除了107个肺结节，其中85个为恶性。病理诊断显示30例MPLC，6例为单原发肺癌。手术过程中的出血量、手术时间、淋巴结清扫数量、术后首日疼痛评分、胸腔引流总量、引流管留置时间以及术后平均住院时间等指标均在可接受范围内，且围手术期无死亡病例。另一研究^[11]^对16例肺癌患者进行同期双侧肺切除术，其中包括12例MPLC患者，所有患者都接受了同期单孔胸腔镜双肺结节切除术，术后没有发生重大并发症及死亡病例，术后康复情况良好。

综上，对于大多数MPLC患者，通常优先考虑手术治疗^[12]^。对于分期较晚或无法承受手术的患者，可以选择放疗、化疗等其他辅助治疗手段。

## 2 局部消融治疗

消融治疗包括射频消融（radiofrequency ablation, RFA）、微波消融（microwave ablation, MWA）、立体定向消融、冷冻消融、激光消融、高强度聚焦超声、不可逆电穿孔术等多种方法^[13]^。这些方法的生理学基础在于通过高温或低温使细胞发生凝固性坏死和死亡。

RFA适用于直径<3 cm的肿瘤，但对于直径>5 cm的肿瘤可能存在消融不完全的风险^[14]^。有研究^[15]^表明，对于医学上不能手术的肺癌患者，RFA治疗的生存率与肿瘤大小密切相关。因此RFA可能是适合高危MPLC患者的一种有前景的替代方法。在这项研究中^[15]^，64例患者接受了亚肺叶切除（n=25）、RFA（n=12）或经皮冷冻消融治疗（percutaneous cryoablation treatment, PCT）（n=27）。研究结果显示，亚肺叶切除、RFA和PCT组的3年生存率分别为87.1%、87.5%和77%，无明显差异。这项研究为RFA作为早期MPLC患者的治疗选择提供了依据，提示RFA在治疗小病灶或早期肿瘤方面的潜力和安全性。一项研究^[16]^招募54例无法手术的IA期肺癌患者进行计算机断层扫描（computed tomography, CT）引导的经皮RFA治疗，1年OS率为86.3%，2年OS率为69.8%。然而这种治疗在肺部肿瘤治疗中也存在局限性，且并发症的发生率较高，如气胸、血胸和支气管胸膜瘘。最近一项研究^[17]^显示，导航支气管镜引导（electromagnetic navigation bronchoscopy, ENB）下RFA是治疗MPLC的一种安全可行的方法，ENB是一种引导支气管镜通过支气管树以准确到达周围病变的技术，利用电磁导航系统协助RFA，极大程度地减少并发症的发生，这项研究展示了在治疗MPLC中应用RFA的可行性和安全性。

与RFA相比，MWA展现出更低的热传导消耗，其局部温度攀升速度更迅猛，且平均消融直径能扩大约25%^[18]^，适合直径>3 cm的肿瘤，尤其是>5 cm的肿瘤，另外，MWA受热沉降效应影响微乎其微，更适合邻近大血管的肿瘤。有研究^[19]^表明，针对肿瘤直径为2-3 cm的早期肺癌患者，采用经皮MWA治疗后，患者1、3、5年生存率分别为97.7%、72.9%、55.7%，同时，治疗死亡率小于1%。有两项研究^[20,21]^发现，MPLC患者接受支气管镜MWA治疗后，完全消融率及2年局部控制率高达70%以上，所有患者的中位PFS约为33个月。因此，对于MPLC患者，除经皮MWA外，经支气管镜MWA也是不错的选择。

在进展期MPLC的治疗中，消融可作为综合治疗的一部分，也可作为一线治疗后的巩固性治疗，或标准治疗失败后的补救措施^[22]^。新兴的电磁导航系统协助RFA可以减少并发症，适用于周围型及中央型肺癌，周围型肺癌或邻近大血管的肺癌更适合选择MWA。肿瘤结节直径≤3 cm适合选择RFA，直径≥5 cm适合选择MWA^[23]^。

冷冻消融通过影像引导精准定位肿瘤病灶，对胶原纤维破坏小，可有效保护血管、支气管等重要结构，尤其适用于靠近肺门或重要解剖结构的肿瘤。冷冻消融过程中，肿瘤细胞会因破裂而释放出肿瘤抗原，这一变化能触发机体的免疫应答机制，进一步提升抗肿瘤效能^[24]^。所以，冷冻消融联合免疫治疗可显著改善患者的免疫功能和近期疗效。

立体定向消融放疗（stereotactic ablative radiotherapy, SABR）将放疗的高剂量精确投照到肿瘤病灶上，使肿瘤受到高剂量照射，而肿瘤周围正常组织受到的照射剂量相对较低^[25]^。SBRT在MPLC中的治疗效果显著，具有高精度、高剂量、短疗程、无创、无痛苦等特点。

相较于单发肺癌，MPLC患者在接受SBRT后的局部控制率、PFS率和OS率方面并没有显著差异^[26]^。一项多中心回顾性研究^[27]^共纳入203例患者，比较了SABR与手术治疗首次接受肺癌根治术的疗效，发现两组治疗3年局部复发率无明显差异。另一项对76例接受SBRT治疗的MPLC患者进行研究^[28]^发现，对于MPLC患者SBRT是一种安全有效的局部治疗替代手术，异时性MPLC（metachronous MPLC, mMPLC）患者接受SBRT治疗后2年OS率接近70%，而sMPLC患者2年PFS率仅为20%，sMPLC患者尽管局部控制良好，但OS和PFS较差，因此sMPLC患者应谨慎接受SBRT治疗。

手术联合SBRT治疗MPLC患者与单独使用SBRT相比具有较好的生存率和生存质量。一项III期临床对照试验^[29]^显示，与标准放疗相比，SABR在有效控制原发灶方面具有显著优势，同时并未加重主要毒副反应。在一项探讨MPLC患者接受多个疗程SBRT后的有效性和安全性研究^[30]^中，有374例患者进行单疗程SBRT，14例患者接受了同步SBRT，48例接受了异时SBRT，108例接受了手术和异时SBRT。接受单疗程患者的3年OS率为54.2%，3年PFS率为67.3%。与单疗程患者相比，仅接受异时SBRT的患者和接受手术+异时SBRT患者的3年OS率分别提升至79.7%和95.4%，3年PFS率分别提高至85.8%和95.4%。而接受同步SBRT的患者与接受单疗程的患者具有相似的3年OS率（46.4%）和PFS率（57.5%）。这项研究表明与单疗程或同步SBRT相比，分期SBRT或手术联合SBRT对MPLC患者疗效更佳。

至于那些无法承受手术或明确拒绝手术的患者，特别是病灶位于中央的MPLC患者，SBRT是一种重要的治疗选择^[31]^。对于手术患者，SBRT可作为辅助治疗，如肺上沟癌首选方案是术前新辅助同步放化疗结合手术切除的综合疗法^[32]^。长期随访研究^[33]^表明，SBRT治疗的MPLC患者可以获得良好的生存率和生活质量。然而，需要更多的研究来确定SBRT在MPLC治疗中的长期效果和最佳治疗策略。

## 3 药物治疗

MPLC的常见药物治疗包括化疗、靶向治疗和免疫治疗，需要根据患者的具体情况进行制定。

化疗可作为术后辅助治疗，也可作为术前新辅助化疗，以达到更好的治疗效果^[34]^，是治疗MPLC的重要手段之一，可以显著改善患者的预后。目前MPLC常见化疗方案有顺铂/卡铂联合卡西他滨/紫杉醇/培美曲塞、吉西他滨联合长春瑞滨等^[35]^。对于MPLC患者，化疗作为术后辅助治疗，对于可切除多发肺癌患者，也可选择含铂双药的术前新辅助化疗，有望达到病理学完全缓解（pathological complete remission, pCR）^[36]^；而有远处转移的MPLC患者，需以化疗为主，联合局部姑息性放疗。化疗在MPLC治疗中也面临挑战，sMPLC的最佳治疗方案尚不明确，手术切除后患者的生存率受到多种因素的影响^[37]^。新辅助化疗适用于可手术的MPLC患者，尤其是肿瘤较大（≥4 cm）或淋巴结阳性的患者。根据AEGEAN试验的结果^[38]^，肺癌患者围手术期使用度伐利尤单抗联合以铂类为基础的新辅助化疗在pCR和PFS方面有显著改善。

靶向治疗主要应用于携带特定驱动基因突变的MPLC患者，常见有表皮生长因子受体（epidermal growth factor receptor, EGFR）突变、间变性淋巴瘤激酶（anaplastic lymphoma kinase, ALK）阳性、c-ros原癌基因1受体酪氨酸激酶（c-ros oncogene 1 receptor tyrosine kinase, ROS1）突变、间质-上皮细胞转化因子（mesenchymal-epithelial transition factor, MET）外显子跳跃等。EGFR突变患者推荐适用EGFR-酪氨酸激酶抑制剂（EGFR-tyrosine kinase inhibitors, EGFR-TKIs），包括奥西替尼、阿法替尼等，ALK阳性推荐克唑替尼、阿来替尼^[39]^。CTONG1103研究结果^[40]^表明，对于局部晚期EGFR突变型MPLC，新辅助靶向治疗+手术+辅助靶向治疗有望成为这类患者更好的治疗选择。一项新的研究^[41]^成果揭示了针对MET 14外显子跳跃突变的靶向疗法，为患者带来新的曙光。当前市面上或即将上市的MET抑制剂琳琅满目，如：克唑替尼、卡博替尼、沃利替尼和特泊替尼等^[42]^，为患者提供了更多选择。这些抑制剂不仅展现出更高的客观缓解率（objective response rate, ORR），并且安全性良好。有研究^[43]^显示，对于至少切除一个EGFR突变病灶的患者，剩余的恶性磨玻璃结节（ground-glass nodule, GGN）（<3 cm）可以接受EGFR-TKIs辅助治疗，靶向治疗应答率为33.3%，ORR为19.7%。一项研究^[44]^证明不同病灶间基因突变的不一致率约为70%-92.9%，意味着单一靶向药物理论上难以全面覆盖所有病灶的治疗需求，需要根据不同药物联合治疗以达到更好的疗效 。

现在，免疫治疗在肺癌治疗领域占据一席之地，尤其在MPLC的治疗中免疫检查点抑制剂（immune checkpoint inhibitors, ICIs）展现出巨大潜力。对于程序性细胞死亡配体1（programmed cell death ligand 1, PD-L1）表达阳性的MPLC患者而言，如帕博利珠单抗等ICIs已成为首选治疗方案之一^[45]^。有研究^[46]^表明，对于某些患者，免疫治疗联合化疗可以提高疗效。一项研究^[47]^纳入21例sMPLC患者，接受信迪利珠单抗治疗10个周期，结果表明sMPLC患者接受ICIs治疗可取得良好的疗效且安全性好。然而，另一项研究^[48]^则对18例接受程序性死亡受体1（programmed cell death 1, PD-1）抗体治疗的肺腺癌患者的37个同步GGN进行分析，发现仅有8.1%的病灶对治疗有反应，而67.6%的GGN病灶无明显变化。也有病例报告^[49]^提示免疫治疗或可作为新辅助治疗方案，但目前无明确证据表明MPLC患者在接受免疫治疗中显著获益。因此ICIs在治疗MPLC中有潜在疗效，但总体争议较大，应用仍面临挑战。

随着免疫治疗的兴起，免疫治疗联合靶向治疗也显示出潜在的疗效^[50]^，还有EGFR-TKIs和化疗的联合使用，但具体的治疗方案和效果需要根据患者的基因特征和肿瘤微环境来制定。化疗仍然是系统治疗的一部分，其在MPLC治疗中的具体作用和效果需要根据具体情况来评估。在药物选择时，除传统基因检测，还可利用液体活检检测循环肿瘤DNA（circulating tumor DNA, ctDNA）或循环肿瘤细胞（circulating tumor cell, CTC），如高灵敏度ctDNA检测、ctDNA甲基化检测等方法，可以预测肿瘤耐药突变和转移风险，从而调整治疗方案，快速识别驱动基因突变，为靶向治疗提供依据，而此外，液体活检可识别与免疫治疗相关的耐药及炎症反应，为MPLC患者的个体治疗提供支持^[51]^。

## 4 多模式治疗

MPLC的治疗是多模式化的，非单一的，综合治疗已成为MPLC的主要治疗方式，且多模式治疗明显提高了患者预后及疗效。本文提到的治疗有手术治疗、消融治疗、药物治疗、手术联合消融、手术联合药物治疗，也包括消融联合药物治疗，其余的治疗方式仍在探索中。有学者提出“Surgey+X”治疗MPLC的模式^[52]^，“X”包括消融、SBRT和靶向治疗，这种治疗模式在治疗早期MPLC中显示出显著疗效和安全性。多模式治疗策略为MPLC患者提供了不同治疗选择。

手术联合消融治疗MPLC是一种新兴治疗方式，这种模式可能包括胸腔镜手术联合ENB的消融治疗，有研究^[53]^报道，10例MPLC患者接受了胸腔镜联合ENB引导消融，术后仅4例出现漏气、肺部感染等并发症，无死亡病例，证明这种联合治疗方法是可行的。另一项研究^[54]^评估了外科切除与MWA联合治疗对于MPLC患者的可行性和安全性。研究结果表明，对于MPLC患者，外科切除联合MWA是一种有效且安全的治疗方法，与单独使用外科切除相比，联合治疗的住院时间和费用更少。

手术切除与SBRT联合使用比较灵活，SABR既可以作为术后对新发病灶或手术未切除病灶的补充治疗，也可以作为术前新辅助治疗，增强机体对肿瘤细胞的免疫反应，提高治疗效果。SBRT联合化疗、靶向、免疫治疗也是可行的，一项研究^[55]^显示新辅助SBRT后序贯替雷利珠单抗联合化疗在治疗肺癌中有较高主要病理缓解率（major pathological response, MPR）和pCR，同时研究放疗联合免疫治疗（I-SABR）对多发肺癌的治疗效果，这项研究是一项开放标签、随机、II期试验，对比了I-SABR与单纯放疗的治疗效果。研究结果显示，I-SABR组合疗法的患者，4年无事件生存（event-free survival, EFS）率高达77%，较仅接受SABR治疗的患者优势显著，提升了24%，联合治疗使患者的疾病复发、远处转移或死亡风险较单独治疗组下降了62%。这表明SBRT联合免疫和化疗可能是一个有效的治疗方法。

手术联合化疗/靶向/免疫治疗是目前临床上MPLC常见的治疗方法。新辅助化疗或术后化疗都是手术的辅助治疗方法，可明显提高切除率并改善患者预后。已有研究^[56]^表明，与单纯新辅助治疗相比，术前使用纳武利尤单抗联合含铂双药化疗预后更好，可以提高手术切除率和pCR，此外，区域性化疗、化疗药物灌注治疗也可以减少术后受治肺的全身效应。研究^[57]^显示，围手术期新辅助免疫治疗显著提升肺癌患者pCR、MPR及PFS，可能有助于提高手术效果，免疫治疗在术后治疗中显示出潜力。胸腔镜联合靶向治疗在MPLC中有一定的应用前景，尤其是EGFR突变阳性患者。相比传统化疗，针对EGFR突变的MPLC患者，靶向治疗作为切除术后的辅助治疗，能够显著改善其预后。

除上述几种联合治疗外，消融联合免疫治疗对非手术切除的患者，可能也是一种选择方法，因为当前免疫治疗的研究是热点，且具有较大治疗潜力。当然，治疗方式不是局限的，除了双治疗联合治疗，多种治疗方式联合也是未来需要探讨的模式。

## 5 总结

综上所述，虽然目前尚未明确MPLC的具体治疗策略，但随着研究的深入，未来可能会进一步优化手术、放化疗、靶向、免疫、消融的顺序和组合，治疗的选择需要根据患者的具体情况来确定，以提高MPLC患者的治疗效果和生存质量。
